# Open Engagement: Exploring Public Participation in the Biosciences

**DOI:** 10.1371/journal.pbio.1000549

**Published:** 2010-11-30

**Authors:** Claire Marris, Nikolas Rose

**Affiliations:** BIOS Centre for the Study of Bioscience, Biomedecine, Biotechnology, and Society, London School of Economics and Political Science, London, United Kingdom

Over the past few decades, numerous initiatives have sought to engage members of the public in decisions concerning bioscience and biotechnologies as early as possible in the development of scientific research, based on the belief that such participation is in the public interest. What these initiatives hope to achieve, however, varies with the motivations for seeking public input. In some cases, they reflect the belief that citizens who will be affected by decisions have the right to participate in those decisions, especially when the research is funded by their tax contributions (this would be what social scientists term a normative justification). In other cases, they reflect a desire to reduce conflict, help (re)build trust, and smooth the way for new innovations (in other words, the reason is instrumental). And in still others, they reflect the assumption that such participation from people who will use and/or be affected by a technology will raise questions about the real-life functioning of developments when they leave the laboratory, perhaps leading to innovations that perform better in complex real-world conditions, or that may be more socially, economically, and environmentally viable (we could term these “substantive justifications”) [1],[2].

Not only do the reasons for soliciting public participation vary, so do perspectives on who might constitute the appropriate public in such endeavours. Should initiatives enlist the input of randomly selected citizens, those with particular interests or kinds of knowledge, those with (or without) strong prior views, those who are specially affected groups such as patients or farmers? For reasons that may be genuine or symbolic, “public engagement” has become almost obligatory in major programmes of publicly funded research aimed at the development of novel biotechnologies.

Current proposals for engaging the public—“participatory Technology Assessment” (pTA, as it tends to be termed in continental Europe) or “upstream public engagement” (as it is commonly termed in the UK)—are, in many ways, a reaction to the widely perceived problems with the Ethical, Legal, and Social Issues (ELSI) approach that was pioneered in the Human Genome Project. The ELSI approach has been criticized for focusing on the social implications of technological developments, without encouraging serious debate about the nature of the developments themselves and the priorities they embody. Public engagement proposals also respond to the widespread perception among politicians and policy makers that there is a “crisis of trust” in the relations between science, politics, commerce, and society. Reflecting this perception, a recent *Nature* editorial [3] suggested using “participatory technology assessment” as a way to achieve improved integration of stakeholder input into decision-making regarding technological innovations. This editorial was prompted by a report, published by the Woodrow Wilson International Center for Scholars in Washington, D.C. [4], that lays out a new vision for US technology assessment, points to recent international experience, particularly in Europe, and calls for a broader, “participatory technology assessment” model that supplements expert opinion with early input from all corners of society.

pTA incorporates several analogous approaches which have been given different names by their respective authors, including: constructive technology assessment [5], interactive technology assessment [6], real-time technology assessment [7], upstream public engagement [8],[9], and technology appraisal [2]. pTA has been implemented through a range of methods, including consensus conferences, citizens juries, stakeholder workshops, deliberative polling, and public dialogues. From our perspective, what is particularly interesting about these initiatives is that they do not focus on making better predictions of public concerns, predicting the potential risks and benefits of a new scientific field, nor do they seek to forestall controversy or to develop ways to manage downstream problems or external outcomes. Rather, they seek to enable a range of actors, including lay publics, but also the widest possible range of people who might be interested or affected, to help shape the trajectory of innovation and, where possible, to keep them open to alternative pathways.

In part, this approach is a recognition of Collingridge's now famous “dilemma of control,” in which “the social consequences of a technology cannot be predicted early in the life of the technology. By the time undesirable consequences are discovered, the technology is often so much part of the whole economic and social fabric that its control is extremely difficult. This is the dilemma of control. When change is easy, the need for it cannot be foreseen; when the need for change is apparent, change has become expensive, difficult and time consuming” [10]. Collingridge argued for the need to develop a “theory of decision making under ignorance”: “Since the future is extremely uncertain, options which allow the decision maker to respond to whatever the future brings are to be favoured.” Of course, such flexibility is difficult to achieve.

Thirty years on, this dilemma, developed in connection to large scale engineering technologies, has become increasingly relevant to the biosciences, where uncertainties as well as potential (positive and negative) stakes for society are particularly high. Political and scientific institutions, troubled by controversies over emerging technologies, most notably GM crops and human embryo research, have developed a new interest in the role that can be played by social scientists in generating public engagement at an early stage in scientific and technological development. pTA, and “upstream engagement”—in which engagement occurs before an innovation has become a fait accompli and pathways of development are still open to debate and scrutiny—have been one dimension of this.

In Europe, such participatory initiatives have been experimented with quite extensively since the early 1990s. They have also been tried to a more limited extent in the US and elsewhere, including in “the global South”—developing and least-developed nations [11]. In the developed world, such initiatives are increasingly seen as the solution to the perceived problem of public mistrust in science and scientists. Yet many scientists still view them with suspicion and do not accept that members of the public with no scientific expertise should be involved in decision-making about scientific matters [12]. Leading scientists are increasingly ready to acknowledge that the public has the right to participate in decisions about the *applications* of science, as recently argued by Lord Rees, President of the UK's Royal Society, Master of Trinity College, and Astronomer Royal [13]. However, this acceptance seldom extends to giving members of the public a role in decisions about the aims, motives, direction, funding, and regulation of scientific research, even though qualitative research on public attitudes by social scientists systematically identifies these questions as crucial for lay citizens' appraisal of developments in science and technology (e.g., [14],[15]).

We should, of course, welcome the new readiness of research funders to build “public attitude” research into many of their big projects at an early stage. Synthetic biology and nanotechnology are examples of areas in which research institutions and their funders are seeking to engage the public, stakeholders and social scientists early on in the development of the field. However, many who are involved with these initiatives—social scientists, representatives of NGOs, members of the public recruited as participants—are sceptical about the value of these “public dialogues.” They often merely describe the beliefs and attitudes of groups comprised of people selected on the basis that they do not have specialised knowledge or strong opinions about the topic in question. And the knowledge gained from these initiatives often seems directed towards anticipating controversy in order to ward it off, rather than to giving the public any actual role in decisions about research trajectories. While some scientific researchers may be wary of involving non-specialists in decisions about the priorities and direction of research, they also need to acknowledge that social factors—beliefs, values, assumptions about what kinds of problems are important to address and what kinds of knowledge might be desirable or useful—are inescapably part of the deliberations of those who shape and fund research priorities in the contemporary world. Genuine and effective public engagement requires us—both life scientists and social scientists—to be more open, more serious, and more inventive in addressing these issues.

This series aims to investigate, through specific case studies, whether, and under what conditions, it is possible to engage the public in scientific issues in meaningful ways in decision-making about the innovation pathways of biosciences. We welcome articles about engagement initiatives that seek to influence the trajectory of scientific research, and the culture of scientific institutions. We particularly welcome articles written by or with scientists who have been involved in such initiatives, describing examples where the diverse participants involved agree that positive outcomes were achieved and so might provide models for further development. In the first article, published today (doi: 10.1371/journal.pbio.1000551), Jean Masson and colleagues describe their experience using interactive technology assessment to solicit input from a broad range of stakeholders for a field trial of genetically modified grapevines in French winegrowing country, in a context—genetically modified crops and winemaking—where resistance to innovation runs deep.

As long-time observers of the processes that influence long-term decisions and institutional structures, we appreciate that retrospective analyses of the outcomes of initiatives that occurred some years ago could be particularly relevant, including those that reflect back on earlier projects for “scientific citizenship” or “social responsibility of scientists.” Contributions to the Public Engagement in Science Series are encouraged; ideas should be sent to plosbiology@plos.org.
